# Supplementary material for: Empirical methods for controlling false positives and estimating confidence in ChIP-Seq peaks
Source: BMC Bioinformatics. 2008 Dec 5;9:523. doi: 10.1186/1471-2105-9-523 (PMC2628906; doi:10.1186/1471-2105-9-523)
Supplement: Additional File 1 — A variety of html documents from the USeq web site detailing the available applications, their best usage, output file type descriptions, command line menus, etc. [file 1471-2105-9-523-S1.zip › USeqUserGuides/JavaDoc.zip!/JavaDoc/bioroot/Feature.html]

Feature


|  |  |  |
| --- | --- | --- |
| **Overview** \| **Package** \| **Class** \| **Use** \| **Tree** \| **Deprecated** \| **Index** \| **Help** | |  |
| **PREV CLASS**   **NEXT CLASS** | **FRAMES**    **NO FRAMES**     **All Classes** |  |
| SUMMARY: NESTED \| FIELD \| CONSTR \| METHOD | DETAIL: FIELD \| CONSTR \| METHOD |  |


---


## bioroot Class Feature

```
java.lang.Object
  bioroot.Feature
```

**All Implemented Interfaces:**: java.lang.Comparable

---

``` public class Feature extends java.lang.Object implements java.lang.Comparable ```

**Author:**
:   nix
    Used to describe a particular feature of a reagent.

---

**Constructor Summary**

`Feature(java.lang.String items)`


| **Method Summary** | |
| --- | --- |
| `int` | `compareTo(java.lang.Object obj)`             For sorting by order. |
| `static java.lang.String` | `createFeatureConcat(Feature[] features)`             Creates a String concatinate from a Feature[] for storage in the database. |
| `static java.lang.String` | `fetchCheckBoxNumberTableRows(Feature[] features, int numColumns)` |
| `java.lang.String` | `getCheckBoxNumber()`             Makes a checkbox and textfield where the box is checked if Feature is visible and a rounded double orderNumber is put in text field if it's not 1000 |
| `java.lang.String` | `getColumnName()` |
| `static java.lang.String[]` | `getFeatureNames(Feature[] features)` |
| `java.lang.String` | `getName()` |
| `double` | `getOrder()` |
| `boolean` | `isVisible()` |
| `static Feature[]` | `makeFeatures(java.lang.String concat)`             Makes a Feature[] from a String concatinate, the thing that is actually stored in the database. |
| `void` | `reset()` |
| `void` | `setColumnName(java.lang.String columnName)` |
| `void` | `setName(java.lang.String name)` |
| `void` | `setOrder(double order)` |
| `void` | `setVisible(boolean visible)` |
| `java.lang.String` | `toString()` |

**Methods inherited from class java.lang.Object**

`equals, getClass, hashCode, notify, notifyAll, wait, wait, wait`

**Constructor Detail**

### Feature

```
public Feature(java.lang.String items)
```


**Method Detail**

### compareTo

```
public int compareTo(java.lang.Object obj)
```

:   For sorting by order.

    :   **Specified by:**: `compareTo` in interface `java.lang.Comparable`

---


### toString

```
public java.lang.String toString()
```

:   **Overrides:**: `toString` in class `java.lang.Object`

---


### reset

```
public void reset()
```

---


### makeFeatures

```
public static Feature[] makeFeatures(java.lang.String concat)
```

:   Makes a Feature[] from a String concatinate, the thing that is actually stored in the database.

---


### createFeatureConcat

```
public static java.lang.String createFeatureConcat(Feature[] features)
```

:   Creates a String concatinate from a Feature[] for storage in the database.

---


### getCheckBoxNumber

```
public java.lang.String getCheckBoxNumber()
```

:   Makes a checkbox and textfield where the box is checked if Feature is visible and a rounded double
    orderNumber is put in text field if it's not 1000

---


### fetchCheckBoxNumberTableRows

```
public static java.lang.String fetchCheckBoxNumberTableRows(Feature[] features,
                                                            int numColumns)
```

---


### getFeatureNames

```
public static java.lang.String[] getFeatureNames(Feature[] features)
```

---


### getColumnName

```
public java.lang.String getColumnName()
```

---


### setColumnName

```
public void setColumnName(java.lang.String columnName)
```

---


### getName

```
public java.lang.String getName()
```

---


### setName

```
public void setName(java.lang.String name)
```

---


### getOrder

```
public double getOrder()
```

---


### setOrder

```
public void setOrder(double order)
```

---


### isVisible

```
public boolean isVisible()
```

---


### setVisible

```
public void setVisible(boolean visible)
```


---


|  |  |  |
| --- | --- | --- |
| **Overview** \| **Package** \| **Class** \| **Use** \| **Tree** \| **Deprecated** \| **Index** \| **Help** | |  |
| **PREV CLASS**   **NEXT CLASS** | **FRAMES**    **NO FRAMES**     **All Classes** |  |
| SUMMARY: NESTED \| FIELD \| CONSTR \| METHOD | DETAIL: FIELD \| CONSTR \| METHOD |  |


---
